# Environmental Risk Factors in Patients with Noninvasive Fungal Sinusitis

**DOI:** 10.1155/2016/5491694

**Published:** 2016-05-04

**Authors:** Badr Eldin Mostafa, Mohammed M. K. El Sharnoubi, Hesham A. A. El-Sersy, Mohammed S. M. Mahmoud

**Affiliations:** Department of Otorhinolaryngology, Head and Neck Surgery, Ain-Shams University Faculty of Medicine, Cairo, Egypt

## Abstract

*Objective.* The aim of our study was to try to determine the possible environmental risk factors for noninvasive fungal sinusitis in Egyptian patients.* Methods.* This is a prospective epidemiological case control study on the environmental risk factors of noninvasive fungal sinusitis. It included 60 patients and 100 age and sex matched controls.* Results.* There was a statistically significant relation between apartment floor, surface area, exposure to dust, exposure to cockroaches, poor air conditioning, and fungal sinusitis. Yet, no statistical significance was found between allergy related occupations, exposure to animals or plants, although their percentages were higher among cases, smoking, and urban or rural residence.* Conclusion.* We suggest that for patients with noninvasive fungal sinusitis a change in their living environment must be implied with better exposure to sunlight, larger well ventilated homes, proper cleaning of dust and cockroach extermination, and if possible the judicious use of air conditioners.

## 1. Introduction

Fungal sinusitis was considered as a rare geographically localized disease until a worldwide increase was reported in the last two decades [1]. Although fungal superinfections are common in immunocompromised individuals, there has been a surge in the affection of immunocompetent hosts [2]. Fungal disease of the nose and paranasal sinuses can be classified based on the clinical, radiologic, and histologic manifestations of the host-pathogen relationship [3]. The most commonly accepted classification system divides fungal rhinosinusitis into invasive and noninvasive diseases based on histopathologic evidence of fungal elements penetrating host tissue. These may be further subdivided into five distinct entities along the immunologic spectrum. Saprophytic fungal infestation (SFI), sinus fungus ball (SFB), and allergic fungal rhinosinusitis (AFRS) are fungal disease manifestations in the absence of fungal invasion of host tissue. Acute fulminant invasive fungal rhinosinusitis (AFIFS), chronic invasive fungal rhinosinusitis (CIFS), and granulomatous invasive fungal rhinosinusitis (GIFS) exhibit histopathologic evidence of hyphal forms within sinus mucosa, submucosa, blood vessels, or bone [4].

The true reasons for this increase are not apparent and may be a combination of environmental factors as well as a higher index of suspicion amongst physicians. The predilection of allergic fungal rhinosinusitis (AFRS) for certain populations revealed some racial [5], socioeconomic [6, 7], and geographical factors [8] or housing differences [9, 10]. The aim of our study was to try to determine the possible environmental risk factors for noninvasive fungal sinusitis in Egyptian patients.

## 2. Patients and Methods

This is a prospective epidemiological case control study on the environmental risk factors of noninvasive fungal sinusitis. It was conducted at Ain-Shams University Faculty of Medicine between January 2013 and June 2014. The study included 60 patients and 100 age and sex matched controls. It was approved by the Local Ethical Committee (Ain-Shams University Faculty of Medicine IRB ENT-7124/6/13).

### 2.1. Group  1: Cases

This group included 60 patients with different types of noninvasive fungal sinusitis suspected based on imaging (heterogeneous opacities, mottling, calcifications, and bone remodeling), intraoperative findings (allergic mucin and fungal debris), and histopathology (PAS staining). Culture (taken intraoperatively) was performed for 42 patients.

Inclusion criteria were as follows:

1. Patients with possible AFS (51 patients).
2. Patients with chronic granulomatous sinusitis (9 patients).
3. Patients with sinus mycetomas.

Exclusion criteria were as follows:

1. Immunocompromised patients.
2. Acute fulminating fungal sinusitis.

### 2.2. Group  2: Controls

One hundred age and sex matched individuals were admitted during the same period of time with no history of nasal or sinus disease or surgery. All had normally appearing nasal cavities and mucosa on nasal endoscopy (a CT scan for controls was considered unethical in this setting).

### 2.3. Methods

A customized questionnaire (asked by a member of the research team, no house visits were carried out) was designed to cover possible demographic variables that may affect the patients, which has the following form:

 Name:
 Age:
 Gender:
 Marital status:
 Number of children:
 Job:
 Address and geographical location:
 Smoking & other special habits:

 Apartment surface area:
 Apartment floor:
 Sun exposure:
 Dust (residential, occupational):
 Air conditioners:
 Exposure to animals (pets, occupational):
 Exposure to cockroaches:
 Exposure to plants (indoors and outdoors):

 Complaint:
 History of present illness:
 Examination:
 CT findings:
 Operative:
 Histopathology:
 Fungal culture:

#### 2.3.1. Statistical Methods

Statistical analysis was performed using IBM SPSS version 22 (IBM Corporation, Armonk, NY, USA). Numerical variables are presented as median and interquartile range and between-group differences were compared using Mann-Whitney *U* test. Categorical variables are presented as number and percentage. Nominal data were compared using Pearson Chi-square or Fisher's exact test when appropriate. Linear-by-linear association is used to compare ordinal data. Multivariate logistic regression analysis was performed to identify independent risk factors for binary outcomes. A two-sided *p* value < 0.05 was considered statistically significant.

## 3. Results

The study involved 160 individuals (60 patients and 100 controls).

### 3.1. Group  1

This group consisted of sixty patients (35 females and 25 males) aged 7–60 years (median 25). There were 51 patients with allergic fungal sinusitis and 9 with chronic granulomatous sinusitis. All patients underwent endoscopic sinus surgery.

### 3.2. Group  2

This group consisted of 100 individuals (54 females and 46 males) with no evidence or history of sinonasal problems. Their age ranged between 6 and 82 years (median 28.5). No statistical significance was found between cases and controls regarding age and gender with *p* values 0.062 and 0.593, respectively.

### 3.3. Variables

#### 3.3.1. Residence

No statistical significance was found by comparing residences whether urban or rural (*p* value, 0.185), while conditions of the residence differed with lower floors (*p* = 0.039) and smaller apartments (*p* < 0.001) and no air conditioners showing statistical evidence of increased occurrence of fungal sinusitis (*p* < 0.001) (Tables 1 and 2).

#### 3.3.2. Smoking

There was no statistical significance (*p* value 0.069) (Table 3).

#### 3.3.3. Exposure to External Factors

Although percentages of cases with poor sun exposure, or exposure to plants, animals, or allergy related occupations, were higher (31.7%, 15.0%, and 36.7%, resp.), there was no statistically significant difference (*p* values: 0.546, 0.250, 0.290, and 0.459, resp.). However, exposure to dust (*p* < 0.001) and cockroaches (*p* < 0.003) was significant (Table 4).

The results of multivariable binary logistic regression analysis for predictors of fungal sinusitis are shown in Table 5. Surface area of residence (*p* value, 0.014), regular exposure to dust (*p* value, 0.006), and poor exposure to AC (*p* value, <0.001) were the only independent risk factors of fungal sinusitis. The model had an adequate overall fit as evidenced by a statistically significant −2 log likelihood test (*p* value, <0.0001) and a statistically nonsignificant Hosmer and Lemeshow test (*p* value, 0.061).

The model had a correct classification rate of 75.6%. The model had a good predictive value as evidenced by an area under the ROC curve (AUC) of 0.85 (95% CI, 0.78 to 0.90). The curve had a sensitivity of 80% (95% CI, 67.7–89.2%) and a specificity of 74% (95% CI, 64.3–82.3%) for a cut-off probability of >0.40 (Tables 5 and 6).

#### 3.3.4. CT Scan

CT scan was performed on all patients and the Lund-Mackay system was evaluation of the degree of sinus pathology. Bone remodeling such as thinning, hypertrophy, and/or expansion were also reported (Table 8).

#### 3.3.5. Laboratory Investigation

CBC were carried out for all subjects, median eosinophil count 400 eosinophils/microL (mildly elevated but statistically insignificant compared to controls). There was no other assessment for allergy.

#### 3.3.6. Fungal Culture

Regarding fungal growth in culture,* Aspergillus* was the most common fungus (25%), followed by* Alternaria* (3%) and* Candida* (1%), while there was no fungal growth in 23% of cases (Table 7).

## 4. Discussion

Although invasive fungal sinusitis is a well-documented disease in immunocompromised patients, many reports have indicated an increased prevalence of fungal sinusitis in otherwise healthy individuals [2].

A worldwide increase in incidence has occurred [1]. This may be due to environmental factors as well as a high index of suspicion among clinicians, microbiologists, and pathologists. The predilection of AFRS for certain populations was not well described until 2004, when a study found African American ethnicity to be a statistically significant predictor of bone erosion in AFRS [7].

Further epidemiologic work demonstrated that AFRS patients were significantly younger than those with chronic rhinosinusitis and were more likely to be uninsured and more likely to live in areas of high poverty or lower median income [5, 6].

Most US cases of AFRS occur within the southern and southeastern geographic regions, where the climate is warm and humid and mold counts are high [8]. Some studies on the health effects associated with self-reported exposure to indoor dampness or mold have found an increase in sinusitis [11]. Moisture, nutrients, and temperature are the most important factors that influence the growth of fungi on buildings [10–12].

In our study, we found that dwelling in lower floors and smaller apartments was associated with a higher incidence of fungal sinusitis. This may be due to more humid environment and less exposure to sunlight. Using the multivariable binary logistic regression model, there was no statistical significance between lower floors and fungal sinusitis. On the other hand, no statistical significance was found between smoking (active or passive) and fungal sinusitis. Similarly, there was no statistical significance between living in urban areas and living in rural areas, although the percentage of cases living in rural areas was higher, maybe due to more exposure to dust, plants, and animals.

Air conditioners (AC) might remove moisture and reduce the relative humidity in rooms. It was found that the average fungal contamination in the house dust of carpets with AC was suppressed by two-thirds compared to rooms without AC. Most probably, fungal contamination is promoted inside the AC but suppressed outside it [9]. Our study confirmed this apparent protective role of AC with a statistically significant value comparing cases and controls.

Exposure to dust is also a significant factor in the development of fungal sinusitis. Dust helps disseminate fungal spores, exposing people to larger inocula. So higher outdoor levels of fungi are associated with concomitant high levels of fungi indoors. Most of the fungi recovered from the inside of houses are external contaminants and not from indwelling organisms [13, 14].

There is evidence that sensitization to cat allergen is associated strongly with allergic rhinitis and asthma [15, 16]. In our study, we did not observe statistical significance between fungal sinusitis and exposure to animals. Although potted plants kept in nonventilated rooms may be a risk factor for the residents since soil may act as a reservoir of fungi [17, 18], we could not find any statistically significant correlation between exposure to plants and fungal sinusitis.

Exposure to cockroach is associated with allergic symptoms and high morbidity in asthmatic patients, and the association of low socioeconomic status and cockroach allergy appears to be independent of age, sex, and race. Several studies have demonstrated that cockroach allergy is found not only in the inner city but also in any substandard housing conditions [19]. Although we found a statistical significance between exposure to cockroaches and fungal sinusitis, using the multivariable binary logistic regression model, there was no statistical significance.

In conclusion, our study showed a statistically significant relation between apartment floor, surface area, exposure to dust, exposure to cockroaches, poor air-conditioning, and fungal sinusitis. Yet, no statistical significance was found between allergy related occupations, exposure to animals or plants, although their percentages were higher among cases, smoking, and urban or rural residence. Although not specifically addressed in the study, there appeared to be no significant difference between socioeconomic levels and the incidence of fungal sinusitis. We suggest that for patients with noninvasive fungal sinusitis a change in their living environment must be implied with better exposure to sunlight, larger well ventilated homes, proper cleaning of dust and cockroach extermination, and if possible the judicious use of air conditioners.

## Figures and Tables

**Table 1:** Condition of residence in cases and controls.

Variable		Cases	Controls	*p* values
Residence	Urban	46 (76.7)	85 (85)	0.185
	Rural	14 (23.3)	15 (15)	

Floor	Ground	14 (23)	8 (8)	0.039
	First	9 (15)	14 (14)	
	Second	14 (23)	26 (26)	
	Third	11 (18)	24 (24)	
	Fourth	6 (10)	18 (18)	
	Fifth	4 (6.7)	7 (7)	
	Sixth or higher	2 (3.3)	3 (3)	

**Table 2:** Median surface area and floor of residence in cases and controls.

Variable	Cases		Controls		*p* values
	Median	IQR	Median	IQR	
Surface	75	65–85	90	80–100	<0.001
Floor	2	1–3	3	2–4	<0.0032

**Table 3:** Smoking status in cases and controls.

	Cases	Controls	*p* values
Nonsmoker	28 (46.7)	34 (34)	0.069
Ex-smoker	0 (0)	7 (7)	
Passive smoker	22 (36.7)	23 (23)	
Active smoker	10 (16.7)	367 (36)	

**Table 4:** Prevalence of risk factors among cases and controls.

Variable	Cases	Controls	*p* value
Occupation	9 (15)	11 (11)	0.459
Poor exposure to sun	22 (36.7)	32 (32)	0.456
Exposure to dust	51 (85)	54 (54)	<0.001
A/C	6 (10)	55 (55)	<0.001
Plants	13 (21.7)	30 (30)	0.25
Animals	19 (31.7)	24 (24)	0.29
Cockroaches	41 (68.3)	44 (44)	0.003

**Table 5:** Multivariable binary logistic regression model for risk factors of fungal sinusitis.

Variable	RC	SE	OR	95% CI	*p*
Age	−0.03	0.02	0.98	0.95–1.01	0.108
Female gender	−0.29	0.4	0.75	0.34–1.64	0.467
Surface of residence	−0.03	0.01	0.97	0.95–0.99	0.014
Dust	1.31	0.47	3.7	1.46 to 9.36	0.006
A/C	−1.87	0.52	0.15	0.06 to 0.42	<0.001
Cockroaches	0.51	0.43	1.67	0.72 to 3.88	0.235
Constant	1.87				

**Table 6:** Model diagnostics.

−2 log likelihood test	
Null model −2 Log Likelihood	211.7
Full model −2 Log Likelihood	154.15
Chi-squared	57.55
DF	7
*p*	<0.0001

Hosmer and Lemeshow test	
Chi-squared	14.92
DF	8
*p*	0.061
Correct classification rate	75.60%

ROC curve analysis	
AUC	0.85 (95% CI, 0.78–0.90)
*p* (AUC = 0.5)	<0.0001
Sensitivity	80% (95% CI, 67.7–89.2)
Specificity	74% (95% CI, 64.3–82.3)

**Table 7:** Fungal growth in culture.

Missed	18 (30.0%)
Nil	23 (38.3%)
*Aspergillus*	15 (25.0%)
*Mucor*	3 (5.0%)
*Candida*	1 (1.7%)

**Table 8:** Lund-Mackay score.

Variable	Allergic fungal sinusitis (*n* = 51)		Invasive fungal sinusitis (*n* = 9)		*p* value
	Median	IQR	Median	IQR	
CT LM score	14	10–20	13	8–16	0.243
